# Participants’ above-chance recognition of own-heart sound combined with poor metacognitive awareness suggests implicit knowledge of own heart cardiodynamics

**DOI:** 10.1038/srep26545

**Published:** 2016-05-23

**Authors:** Ruben T. Azevedo, Salvatore Maria Aglioti, Bigna Lenggenhager

**Affiliations:** 1 IRCCS Fondazione Santa Lucia, Via Ardeatina 306, 00100 Rome, Italy; 2 Department of Psychology, University of Rome “La Sapienza”, Via dei Marsi 78, 00185 Rome, Italy

## Abstract

Mounting evidence suggests that interoceptive signals are fundamentally important for the experience of the self. Thus far, studies on interoception have mainly focused on the ability to monitor the timing of ongoing heartbeats and on how these influence emotional and self-related processes. However, cardiac afferent signalling is not confined to heartbeat timing and several other cardiac parameters characterize cardiodynamic functioning. Building on the fact that each heart has its own self-specific cardio-dynamics, which cannot be expressed uniquely by heart rate, we devised a novel task to test whether people could recognize the sound of their own heart even when perceived offline and thus not in synchrony with ongoing heartbeats. In a forced-choice paradigm, participants discriminated between sounds of their own heartbeat (previously recorded with a Doppler device) versus another person’s heart. Participants identified the sound of their own heart above chance, whereas their metacognition of performance – as calculated by contrasting performance against ratings of confidence - was considerably poorer. These results suggest an implicit access to fine-grained neural representations of elementary cardio-dynamic parameters beyond heartbeat timing.

It is now held that the ever-continuous representation and regulation of bodily signals provide the foundations for the experience of the self^ ^1. We exist in our bodies, as our bodies exist in our minds^2^. Traditionally, experimental work in this field has focused on the processing of exteroceptive (e.g. tactile and visual) and motor bodily signals. Many experimental paradigms have been developed over the last years to manipulate these signals and to investigate their influence on the bodily self^3^,^4^,^5^,^6^. However, the influence of the private internal body, i.e. interoceptive signals^7^, has been largely overlooked and has only rather recently entered the experimental study of the self 8,9,10. This is potentially surprising, as it has long been argued theoretically that self-awareness emerges from an image of the homeostatic state of the body, i.e. from the constantly present interoceptive signals^11^,^12^. The reason for this dearth of experimental research may be that inherently private interoceptive sensations are not easily amenable to experimental measurement and manipulation. Cardiac sensations provide a privileged source of information for the study of interoception. The central role of mapping, re-mapping and interpreting one’s own cardiac signals has long been recognized in theories of emotion^11^,^12^. Yet people differ greatly in their ability to explicitly and accurately identify discrete interoceptive events such as heartbeats^13^. This ability, often named interoceptive accuracy (IAcc;^14^) is typically assessed by asking participants to track their heartbeats for short periods of time^13^, or by asking them to discriminate between auditory tones presented either synchronously or asynchronously with their heartbeats^15^. While individual differences in IAcc have been shown to predict a wide number of cognitive and emotional processes^16^, it is widely accepted that the influence of interoceptive signals in, for example, emotional processing^17^,^18^,^19^,^20^, or in the sense of the self^21^,^22^,^23^, often occurs without full explicit awareness. Indeed, people demonstrate remarkable different levels of *awareness* of their success when performing interoceptive tasks (i.e., their meta-cognitive awareness differs). In other words, their ability to identify interoceptive signals, i.e. heartbeats, is not always paralleled by accurate judgments of their own performance^14^,^24^. This lack of explicit awareness, or meta-awareness, makes the study of interoception additionally challenging but also appealing.

Thanks to new experimental paradigms, recent studies indicate that interoceptive signals are constantly integrated with exteroceptive (i.e. visual or auditory) signals - even if often at an implicit level - in order to build a coherent representation of the own body^21^,^22^. For example, Aspell and colleagues^21^, presented participants with a virtual body whose silhouette could be flashing either synchronously or asynchronously with respect to their own heartbeats. Even if participants were not aware of this contingency, they identified themselves more with the avatar in the synchronous condition. In a similar line of research, a recent electrophysiological study has shown sensory suppression of heartbeat-related auditory tones - again without the participants’ explicit awareness^23^. Both studies suggest that cardiac signals presented through an exteroceptive channel can activate and be compared with representations of interoceptive information (see also^25^). This implies that the bodily awareness is, at least partially, grounded in a successful integration of extero- and interoceptive signals.

Importantly, however, the vast majority of research into interoceptive awareness as well as into implicit integration of intero- and exteroceptive signals, has focused on cardiac timing (i.e. the timing of the occurrence of each heartbeat). Yet cardiac afferent signalling is not confined to simple heartbeat timing. Several other cardiovascular parameters of autonomic reactivity^26^ as well as parameters related to cardiac inotropy^27^ have been shown to mediate interoception. Indeed the various cardiac parameters are interrelated and it is their concerted activity that fully characterizes cardiovascular functioning and regulation. It is known that each heart has its own self-specific cardio-dynamics, which cannot be expressed uniquely by mere heart rate. Such a “cardiac fingerprint” is detectable by computer algorithms that can successfully recognize individual hearts independently of their heart rate at the moment of recording^ ^28. Accordingly, in order to comprehend the various parameters comprising one’s own cardiac signature, a person’s representation of their cardiac information will not be restricted to the online processing of the timing of their heartbeats. It is worth noting here that in other domains, e.g. locomotion, it has been shown that people not only are able to monitor their movements online but can also recognize their “motor signature” when asked to recognize their movement on a point light walker^29^,^30^ as well as on a virtual character^31^ from a third person perspective via offline visual presentation. Moreover, such recognition seems to rely on an individual’s implicit knowledge about the intrinsic temporal dynamics of his/her movements, which has been gained, at least partially, through a lifetime of afferent proprioceptive and sensorimotor experiences^31^.

In this study, we aimed to explore whether people have implicit and/or explicit knowledge of their cardiac signature, i.e. whether people can discriminate their own self-specific cardio-dynamics, even when presented offline. In a novel forced-choice paradigm (see Fig. 1), we asked participants to discriminate between sounds of their own heartbeat previously recorded (with a Doppler device, see below) and those of another person’s heart. We have previously shown that presenting these heart sounds online to participants can implicitly modulate their behaviour^20^, but it remains unclear if participants can actually recognize the sound of their own heart. To control for the possibility that own-heart recognition could be merely due to cardiac timings or to a general knowledge about their own heart rate, the samples of the other-heart sound were matched in heart-rate. If participants are able to identify their own cardiac signature, we expected them to perform significantly above chance. In order to assess whether performance is underpinned by implicit or by explicit processes^32^, participants were further asked to rate their confidence in their decisions after each trial. Comparing objective performance with subjective perceptions of the own accuracy allowed us to calculate participants’ (meta) awareness of their ability to recognize their own-heart sounds^14^. A heartbeat counting task^13^ was further used to enable us to relate the participant’s ability to recognise their own-heart sound recognition to a standard measure of interoceptive accuracy.

## Results

### Recognition of the sound of the heart

Participants’ performance was analysed using binominal distribution probabilities, which indicate that performance above chance would be shown by correct recognition of 20 trials, out of the total 30 trials (p = 0.028).

**Figure 2:**
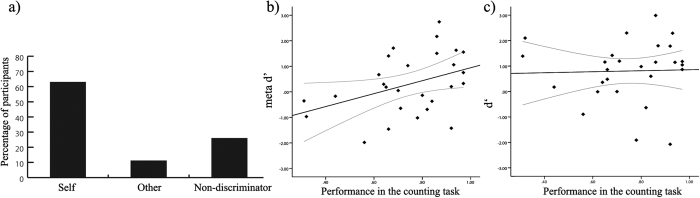
Results: (**a**) Percentage of participants grouped according to their performance in the sound recognition task; (**b**) Correlation between performance in the counting task and meta-awareness; (**c**) Correlation between performance in the counting task and performance in the sound recognition task.

Accordingly, participants were assigned to three groups (see Fig. 2a): 1) the SELF group contained those participants who had accuracy rates above 0.67, i.e. 20 or more trials correct, and therefore performed significantly better than chance. In other words, these participants correctly attributed their own heart to themselves; 2) the NON-DISCRIMINATOR group comprised those participants who performed at chance level (i.e. accuracy rates between 0.33 and 0.66); 3) the OTHER group was made up of those individuals who performed significantly below- chance (accuracy below 0.33). These participants incorrectly identified the other person’s heart as their own heart.

The distribution of participants between the different groups is shown in Fig. 2a. 17 out of 27 fell into the SELF group (i.e., correct self-attribution, mean accuracy 0.77 (SEM 0.02), see Table 1), 7 out of 27 fell into the NON-DISCRIMINATOR group (i.e., they responded at chance-level (mean accuracy 0.52 (SEM 0.03)) and 3 out of 27 fell into the OTHER group (i.e., misattribution of the other’s heart as their own heart, mean accuracy 0.21 (SEM 0.06)). A chi-square test suggests significant differences in numbers between the groups (chi square = 11.6, p = 0.003, Cohen’s ω = 0.65). Post-hoc chi square tests revealed that more participants belonged to the SELF group than to the NON-DISCRIMINATOR (chi square = 4.2, p = 0.04, ω = 0.42) and OTHER groups (chi square = 9.8, p = 0.002, ω = 0.70). The numbers in the two latter groups did not differ significantly.

### Other measures

#### Confidence ratings

Confidence ratings in the experimental condition was an average of 43.2 (SEM 3.5, ranging from 4.9 to 64.5). The three groups did not significantly differ in their confidence ratings (Kruskal-Wallis, chi square = 0.53, p = 0.77, ω = 0.14).

#### Heartbeat Counting task

The performance in the heartbeat counting task was on average 0.75 (SEM 0.04, ranging from 0.31 to 0.97). The three groups did not significantly differ in their performance (Kruskal-Wallis, chi square = 3.37, p = 0.18, ω = 0.59).

#### Meta awareness

Participants’ meta-awareness of performance (meta-d’) was calculated as a function of objective performance and associated confidence ratings, according to “type 2” Signal Detection Theory indices of sensitivity^32^. Meta-d’ scores (Mean 0.30; SEM; 0.23) were significantly lower (t = 2.6, p = 0.016, Cohen’s d = 0.43) than d’ (Mean 0.80, s.d. 0.23) showing that participants had poor awareness of their own accuracy, which suggests intuition-based behavior. Moreover, correlation analyses revealed that meta-d’ scores (r = 0.40, p = 0.041) but not d’ scores (r = 0.03, p = 0.88) were significantly correlated to performance in the heartbeat counting task. The former correlation is larger than the latter: t = 2.19, p = 0.028, using Lee & Preacher, test for dependent correlations^33^; see Fig. 2b,c.

### Control task

Analysis of accuracy was performed identically as in the experimental task. 3 out of the 16 participants fell into the SELF group (mean accuracy 0.87, see Table 1), 11 into the NON-DISCRIMINATOR group (mean accuracy 0.49) and 2 into the OTHER group (mean accuracy 0.13). A chi square test suggests a significantly different distribution between the groups (chi square = 9.13, p = 0.01, ω = 0.58). Post-hoc chi square tests suggest that more participants belonged to the NON-DISCRIMINATOR group than to the SELF group (chi square = 4.6, p = 0.03, ω = 0.44) and OTHER group (chi square = 6.2, p = 0.01, ω = 0.79), while the two latter groups did not differ significantly from each other (chi square = 0.2, p = 0.66, ω = 0.1).

### Debriefing

In the semi-structured debriefing interview all participants reported believing that the sounds belonged to 3 or more different hearts. They further reported that their choices were mostly based on guessing or on intuition. Even the few participants who felt considerably confident in having discriminated their own heart correctly still confessed difficulties in identifying the criteria used. Together this evidence supports the finding that the identification of own-heart sound was performed implicitly.

## Discussion

Bodily signals, particularly those arising from the interoceptive system, are thought to set the foundations on which the subjective experience of the self unfolds^2^,^9^,^10^. This notion of embodied self has received convincing support from recent empirical research, which has shown that that cardiac signals are integrated with external information in order to build a coherent sense of self 21,22,34. Here, we aimed to address a previously neglected dimension of interoception, namely the offline representation of the inner body. Specifically, we explored whether healthy participants were able to recognize their own cardiac signature, independent of their current interoceptive state, i.e., whether there was offline recognition of self-specific cardiovascular dynamics. Our method was first to record the participants’ heart sound with a Doppler device and then to ask them to discriminate the sound of their own heart from that of someone else’s heart. The study had two main findings. Firstly, most participants performed significantly above chance, indicating that participants were indeed able to discriminate between previously recorded, and thus offline, sounds of their own and another person’s heart. Importantly, this finding could not be based merely on heartbeat timing because this parameter was matched between the participant’s own and the other person’s heart sound. Moreover, the effect was absent in the control condition, where acoustic tones were delivered in synchrony with the participant’s own pre-recorded heart beats. Our findings extend recent empirical research which has indicated that participants integrate externally presented (online) auditory or visual cues with cardiac signals during body-related processing^21^,^22^,^23^. We show that a representation of an individual’s cardiac signature goes beyond mere heartbeat timings. Furthermore, our results also suggest that people can, at least partially, access and translate neural representations of elementary cardio-dynamic parameters that have been formed throughout time (probably through sensory, acoustic or mechano-pressure mechanisms) and can compare these representations with externally presented auditory stimuli (as the sound of the blood flow recorded with a Doppler device). Even though participants could not recognize the criteria they were using, their performance indicates that they were able to identify the sounds that matched their cardiodynamic signature. So far we can only speculate about the underlying mechanisms. However, we believe that they may be comparable to those that underpin the comparison of proprioceptive and sensorimotor representation of own movements when these are presented offline, such as gait^29^; skilled actions^35^; or facial motion^31^. Importantly, this ability is thought to rely not merely on previous first-person visual experiences, but principally to involve the proprioceptive and sensorimotor feedback from previous movements^31^. In keeping with models that have extended (from exteroceptive to interoceptive perception) the notion of comparing different types of bodily representations^10^, we show in this experiment that the interocpetion is likely to be grounded both on online and offline body representations of the inside of our body (interoceptive representations). The continuous mapping and re-mapping of afferent signals form and maintain accurate representations of the body’s structural properties, as well as of the fine grained dynamic processes that regulate its functioning. Holding coherent and detailed maps of the body allows the brain to efficiently detect, simulate and regulate changes in its state, in order to maintain homeostasis^36^.

The second result of our study is that the recognition of one’s own heartbeat signature seems to be based mostly on implicit processes. Indeed, not only did participants report intuition-based behaviour but their objective performance on the forced-choice task clearly exceeded their metacognitive awareness. Thus, although their behaviour indicates recognition of their own heart sound, participants were less good at judging their ability to perform the task itself. This indicates that the perceptual decision about self vs other heart sound was, at least partially, guided by implicit processes^32^,^37^. Tellingly, only metacognitive awareness was correlated to the classical interoceptive accuracy measure that we tested (i.e., accuracy in heartbeat counting^13^). The results of the correlation analyses suggest that even if, contrary to what could be expected, individuals with high interoceptive accuracy were no better at discriminating the sound of their heart, they were more aware of their ability to perform the task. Given the relatively small sample size, these results must be treated with caution. However, we believe that they: i) suggest that offline and online representations of interoceptive signals may rely on different neurocognitive underpinnings; and ii) add to recent literature which has shown a dissociation between the various dimensions of interoception, such as interoceptive accuracy and interoceptive awareness (i.e. meta-cognition^14^,^24^,^38^,^39^). In our study, individuals with high interoceptive accuracy seem to rely in a greater extent on explicit criteria to guide their decision^32^,^37^. What this suggests is that good interoceptors, who presumably hold more accurate representations of their online bodily states, are also more aware of their elementary bodily dynamics. Even if they did not have higher ability to discriminate their own-heart sound, they showed better appraisal of their decisions, arguably because they relied on a coherent understanding of their ability to perceive bodily signals.

Several limitations of this study should be considered. *First*, the study was not designed to identify the individual differences in cardio-dynamic parameters (e.g. stroke volume, ejection time), which could have contributed to accurate discrimination of own-heart sound. Future research should aim at identifying such parameters, for example by recording longer samples using equipment that allows the extraction and estimation of cardiovascular parameters (e.g., impedance cardiography). Likewise, the interoceptive pathways (e.g. tactile somatosensory, mechanoreceptors within the heart) and mechanisms underlying cardiovascular perception and the ability to recognize own-heart sound have yet to be determined. *Furthermore*, we did not assess factors that are generally known to influence interoceptive accuracy such as for example gender, mean heart rate^40^,^41^ or BMI^42^.

Nonetheless, we believe that this study represents a significant conceptual step and likewise a departure from previous rather simplistic heartbeat-timing-dependent approaches to cardioception. We suggest that research into interoception, both in the context of its contribution to the bodily self as well as to various cognitive and emotional processes, would benefit from acknowledging the importance of considering the full complexity of cardiovascular activity^27^ and the need to adopt new methodologies to study cardiac interoception^20^.

## Methods

### Participants

Twenty-eight healthy participants took part in the main study. A participant who was a medical doctor (n = 1) was excluded from the analyses, as a theoretical knowledge on cardio dynamics or experience in discriminating heart beat sounds could influence performance. The final sample size thus comprised twenty-seven participants (19 female, mean age 28.6 +/−3.3 STD), none of whom had previously heard their own-heart sound as translated by a doppler device. All participants gave written informed consent. The study and all experimental procedures were approved by the ethics committee of the IRCSS, Fondazione Santa Lucia and were carried out in accordance with the ethical standards of the 1964 Declaration of Helsinki.

A sub-group of these participants (n = 16, 8 female, mean age 30.3 +/−3.4 STD) completed the additional control task (see below).

### Experimental procedure

#### Stimuli

The participant’s heart rate was recorded during one minute using a pulse transducer (Adinstruments; TN1012/ST), which was attached to the index finger using a velcro fastener. Simultaneously, a Doppler device (Angel sound, Fetal Doppler, http://www.jumper-medical.com/) placed over the participant’s heart to record the sound of the heart. The Doppler detects blood flow and generates a sound reflecting its dynamics (e.g. varying intensity (loudness), frequency (pitch), quality and duration). Because the direction and velocity of blood flow is different throughout the cardiac cycle, the audible sound represents both diastolic filling and systolic ejection^43^. Several individual cardiovascular parameters can be inferred from the Doppler sound by a trained person, or objectively extracted by computational algorithms. For example, the first heart sound (i.e. caused by the closure of the tricuspid and mitral valves) and second heart sounds (i.e. caused by the closure of the aortic and pulmonary valves) can be perceived^44^,^45^. Moreover, additional parameters related to cardiac inotropy (e.g. ejection velocity, stroke volume) and diastolic/systolic relations can be extracted^43^. The recorded sound of the heart was treated with a sound processing software (Ableton Live 8.2.2) to reduce noise using low-pass frequency filters (1.35 kHz) and set the sound to a standard volume. This recording was automatically cut into ten 5s-samples using a computer algorithm. The *own heart samples* consisted on these ten 5s samples, five of which were repeated twice. The *other person’s samples* consisted on ten 5s samples of another person (again five of these samples were presented twice), individually matched in terms of heart rate. The mean difference in heart rate between the two samples was 1.47 bpm (SD 1.54).

#### Experimental task

Participants were seated comfortably with headphones on. They were asked to fixate the screen and concentrate on the sound samples presented over the headphones. They were told that they would be listening to various short audio clips of heartbeat sounds. Eprime (Psychology Software Tools, Pittsburgh, PA) was used to randomly present the pre-recorded samples of their own or another person’s heart sounds. A total of 30 5-s samples were presented, which contained the presentation of 15 samples of their own and 15 samples of another person’s heartbeat sounds. After each sample, the participants indicated in a forced-choice task whether the heard sound had reproduced their own heart, or not. It was explicitly explained that the presented sounds were offline. After each response they rated on a VAS scale ranging from 0 (not confident at all) to 100 (highly confident) their confidence in their decision. A sub-group of the participants further completed the control task (see below), which was added to the experiment in order to test whether self-recognition of one’s own heart could be based on the recognition of heartbeat timing, for example, heart rate variability. In those participants the experimental and control task were carried out in counterbalanced order across participants.

#### Measure and calculation of interoceptive accuracy

Interoceptive accuracy was measured prior to the experimental task using the “heartbeat counting paradigm”^13^. Participants internally counted their heartbeats during four intervals (25s, 35s, 45s, and 100s) presented in random order. The start and end of the period was signalled by an auditory cue delivered by Eprime via the headphones. The real heart rate was recorded using a pulse transducer (Adinstruments; TN1012/ST), which was attached to the index finger using a Velcro fastener. All participants reported that they could not feel their pulse on the finger. These two measures were used to calculate an accuracy index as described in^13^.

#### Measure and calculation of metacognitive ability (Meta-d’)

We combined confidence ratings with objective performance in order to estimate participant’s metacognitive ability. Classical (or Type I) signal detection theory (SDT) measures quantify the subject’s ability to discriminate (d’) between the presence (e.g. own-heart sound) and the absence (e.g. other-heart sound) of a stimulus, independently of responses biases, i.e. the tendency to favor one type of response over the other^46^. d’ can be calculated as d’ = z(Hit rate) − z(false alarm). These measures of objective performance can be extended to evaluate the ability to discriminate between one’s own correct and incorrect answers, i.e. metacognitive ability (type 2 SDT)^46^,^47^. A close correspondence between confidence and accuracy (e.g. correct trials accompanied by high confidence ratings or wrong trials accompanied by low confidence ratings), indicates that the participants have good knowledge on the correctness of their responses (i.e. a high metacognitive ability). Several SDT-based measures have been proposed to quantify metacognition ability. Here, we used a recently developed measure (meta-d’; sum-square error approach) to calculate type 2 SDT^32^ (http://www.columbia.edu/~bsm2105/type2sdt/). Importantly, unlike standard type 2 SDT measures^47^, meta-d’ is robust to response biases and type I performance^32^,^37^. In other words, meta-d’ scores are reasonably independent of the tendency to provide one answer type over another (e.g. “my heart” vs “not my heart”) as well as the ability to correctly discriminate one’s own-heart sound. Moreover, meta-d’ and d’ are expressed on the same scale and therefore can be directly compared to provide an estimate of how much subjects base their decisions on explicit knowledge^32^,^37^. If metacognition were to be optimal, the subjects would be expected to make use of all the information available for the type I task when judging their own performance, with the consequence that meta-d’ = d’. Conversely, in sub-optimal metacognition conditions meta-d’ < d’. In that case, because subjects are not able to judge the correctness of own response (i.e. provide high-confidence ratings to incorrect responses and low-confidence ratings to correct responses), it is believed that they were not (fully) aware of the criteria used to guide their performance. Thus, their performance will have been (at least partially) underpinned by implicit processes.

A similar approach has been previously used to estimate metacognitive awareness of interoceptive accuracy in classical measures of interoception (ref. 14). However, in the current study we adopted the standard protocol of the heartbeat counting task, which is comprised by only four trials and is therefore not ideal to estimate a reliable index of metacognitive ability.

### Control task

A control task was designed to understand a possible contribution of pure timing effects (i.e. heart rate frequency and variability). The procedure of stimuli creation followed the procedure described above but instead of recording the Doppler sound we used a hardware-based function of Powerlab (www.adinstruments.com) to detect the peak of the finger pulse and at each pulse trigger the presentation (Eprime; Psychology Software tools) of a standard tone (1000 Hz, 100 ms). These tones were recorded using Audacity (http://audacity.sourceforge.net/). Then the own and other person’s heart sounds were again cut in 5s samples, which were presented as described above.

### Debriefing

At the end of the experiment, participants were verbally asked in a semi-structured way, to report how many different hearts they thought the audio samples had been obtained from, as well as how difficult they perceived the different tasks to be. The debriefing was included as a complement to confidence ratings, in particular in order to understand if i) participants felt confident about their performance in this task, i.e. if they felt they could identify the sound of their own heart and if yes, based on which subjective criteria and ii) if they could figure out how many different heartbeats there were.

## Additional Information

**How to cite this article**: Azevedo, R. T. *et al.* Participants’ above-chance recognition of own-heart sound combined with poor metacognitive awareness suggests implicit knowledge of own heart cardiodynamics. *Sci. Rep.* **6**, 26545; doi: 10.1038/srep26545 (2016).

## Figures and Tables

**Figure 1:**
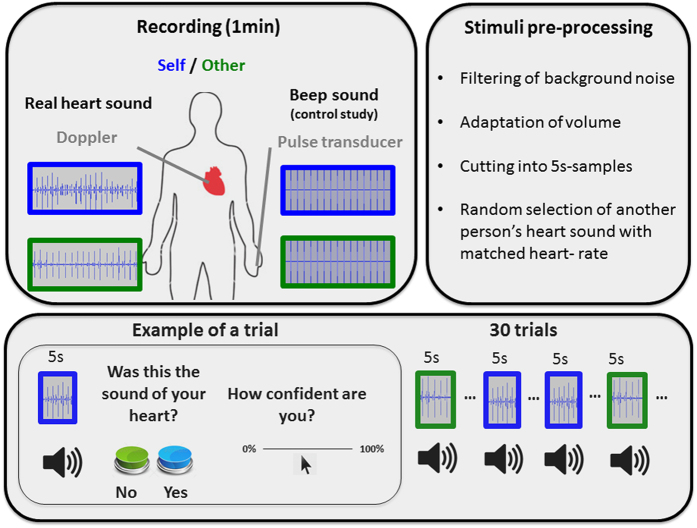
Experimental procedure: Before the experiment started, we recorded: (a) the sound of the participant’s heart using a Doppler device (main experiment); (b) and “beep” sounds triggered by the participant’s pulse waves measured with a pulse transducer (control experiment). After pre-processing, short samples of their own or another person’s heart sounds were presented, in random order. After each of the 30 trials, participants had to make a forced-choice decision about whether they had heard the sound of their own heartbeats or not, followed by a rating of their confidence in their decision.

**Table 1:** Numbers and percentages of participants falling into the different groups in the experimental task.

Group	Self	Non-discriminators	Other
Heartbeat	17/27 (63%)	7/27 (26%)	3/27 (11%)
Beep	3/16 (18.75%)	11/16 (68.75%)	2/16 (12.5%)

Upper row using real heartbeat sound; lower row in the control task using R-wave evoked beep sounds.
